# The Hidden Triad: Decoding Zinner Syndrome Through Computed Tomography Urography and Magnetic Resonance Imaging

**DOI:** 10.7759/cureus.91160

**Published:** 2025-08-28

**Authors:** Sooraj Narayan, Balaji Kombde, Atish Komwad

**Affiliations:** 1 Department of Radiology, Government Medical College, Latur, Latur, IND

**Keywords:** computed tomography-urography, magnetic resonance imaging, renal agenesis, seminal vesicle cyst, zinner syndrome

## Abstract

Zinner syndrome is an uncommon congenital anomaly involving the triad of unilateral renal agenesis, ipsilateral seminal vesicle cyst, and ejaculatory duct obstruction. It is often asymptomatic until puberty or adulthood, when patients may present with genitourinary complaints.

We report the case of a 17-year-old male presenting with mild suprapubic discomfort and occasional right testicular pain. Clinical examination was unremarkable. Computed tomography (CT) urography revealed right renal agenesis with a large paravesical cystic lesion. Magnetic resonance imaging (MRI) of the pelvis confirmed a tubular, hyperintense cystic structure adjacent to the prostate and seminal vesicle, containing incomplete septations. These findings were consistent with a seminal vesicle cyst, confirming the diagnosis of Zinner syndrome. Radiological imaging - particularly MRI and CT urography - plays a pivotal role in accurate diagnosis and preoperative assessment. Awareness of this condition and its imaging features is essential for timely identification and management.

## Introduction

Zinner syndrome is a rare congenital malformation, comprising a triad of unilateral renal agenesis, ipsilateral seminal vesicle cyst, and ipsilateral ejaculatory duct obstruction [1,2]. It arises due to abnormal development of the mesonephric (Wolffian) duct during embryogenesis. The incidence is reported to be 0.046% [1]. Most patients remain asymptomatic until the second or third decade of life, when symptoms related to obstruction or infection manifest. Presentations include perineal discomfort, painful ejaculation, dysuria, epididymitis, prostatitis, or infertility [3-5]. Diagnosis is often delayed due to nonspecific symptoms and requires a high index of suspicion, supported by imaging.

## Case presentation

**Figure 1:**
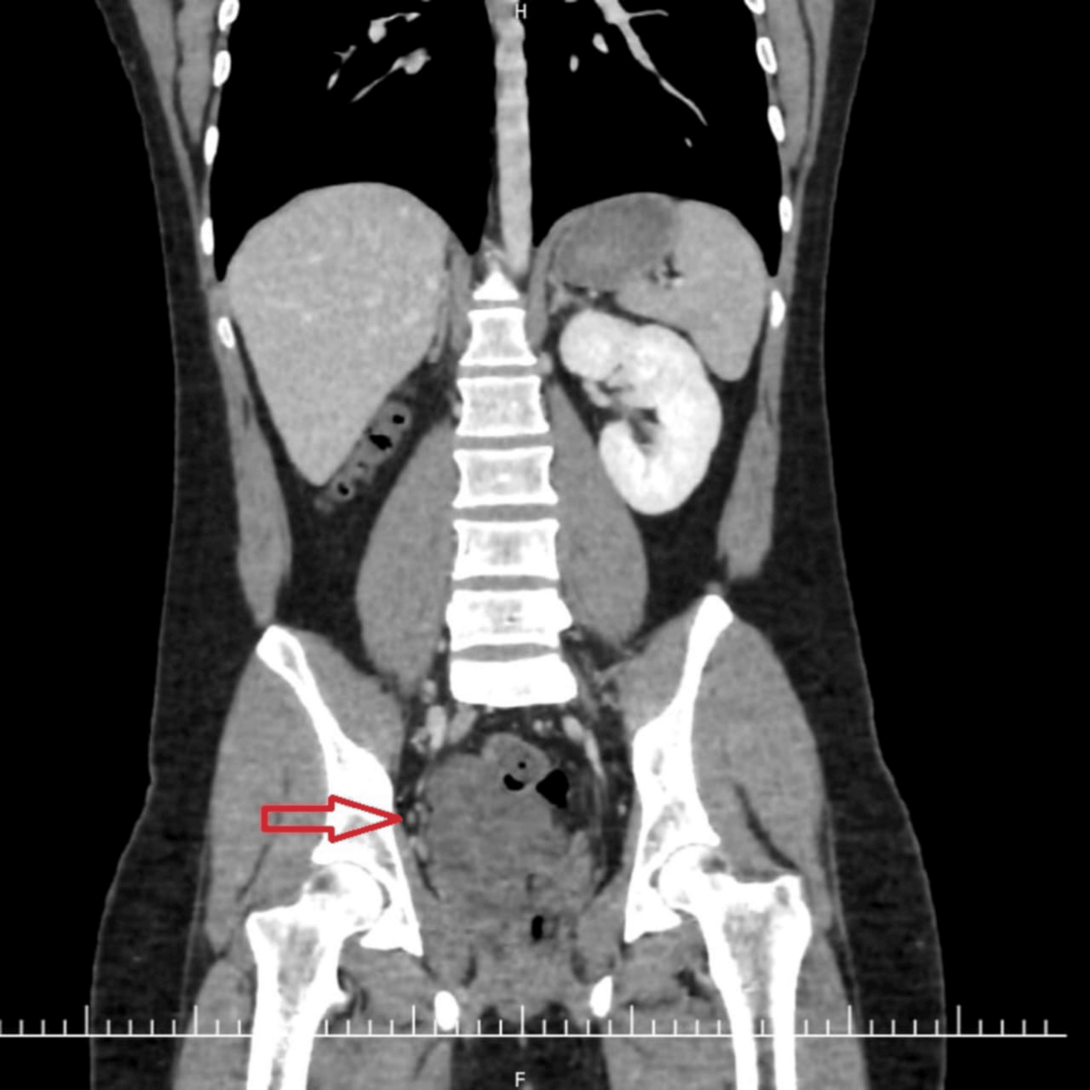
Coronal contrast-enhanced CT urography image shows complete absence of the right kidney and a large, well-defined, septated cystic lesion in the right paravesical space, suggestive of a seminal vesicle cyst (arrow). CT, Computed tomography

**Figure 2:**
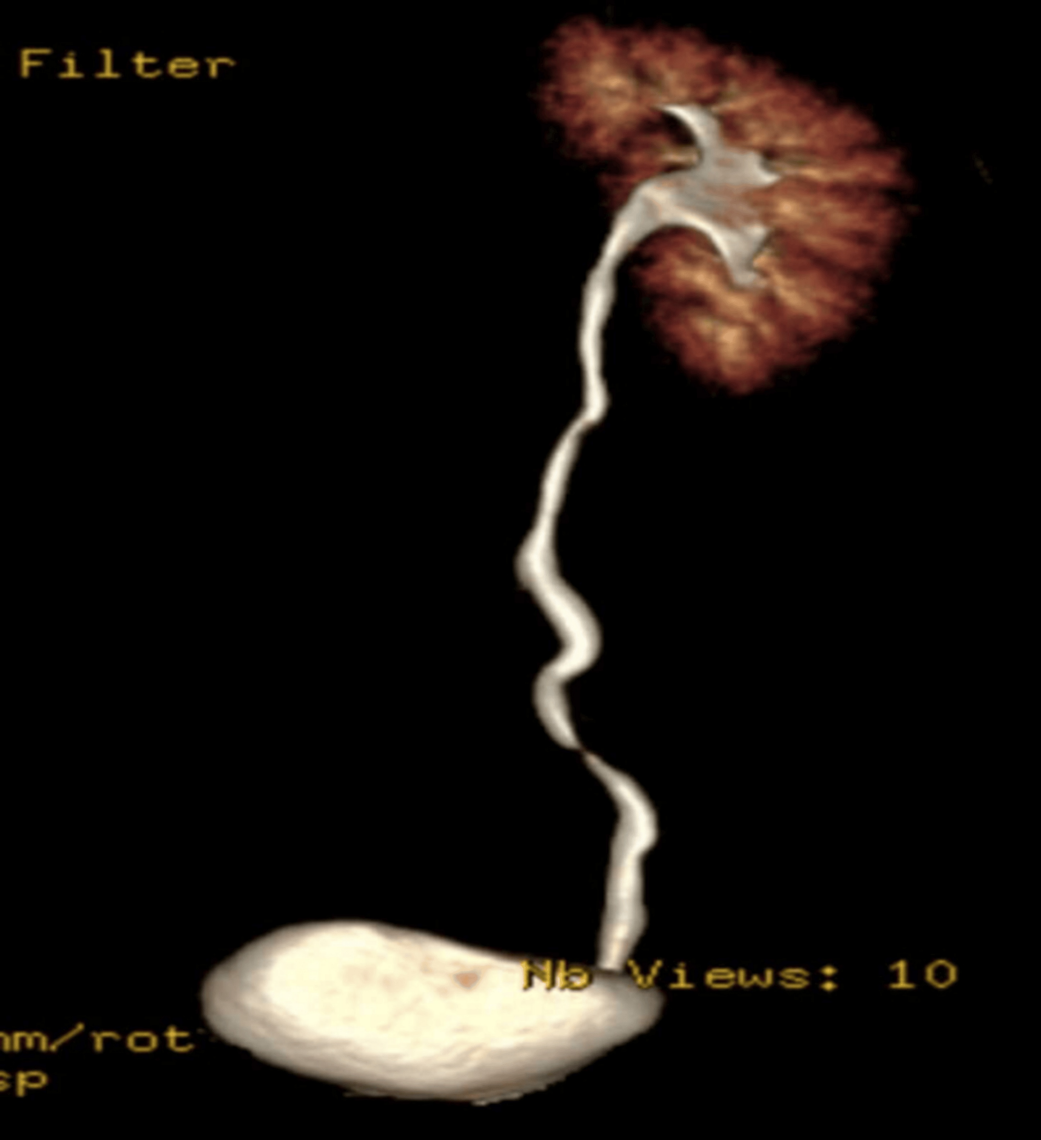
Coronal 3D CT urography image shows right renal agenesis. CT, Computed tomography

A 17-year-old male presented with complaints of intermittent mild suprapubic discomfort and occasional right testicular pain for several months. There was no history of urinary tract infection, hematuria, fever, or dysuria. The patient had no previous history of genitourinary interventions or surgeries. On clinical examination, both testes were normally descended, with no tenderness or masses. Digital rectal examination was within normal limits. Laboratory investigations, including complete blood count, renal function tests, and urinalysis, were normal. Computed tomography (CT) urography with 3D reconstruction was performed as the initial imaging modality, revealing an absent right kidney with compensatory hypertrophy of the left kidney (Figures 1-2). Additionally, a well-defined, tubular, thin-walled cystic lesion with incomplete septations was noted in the right paravesical space, suggestive of a seminal vesicle cyst (Figure 1). No calcification or ureteral remnants were seen. These findings prompted further evaluation with magnetic resonance imaging (MRI).

**Figure 3:**
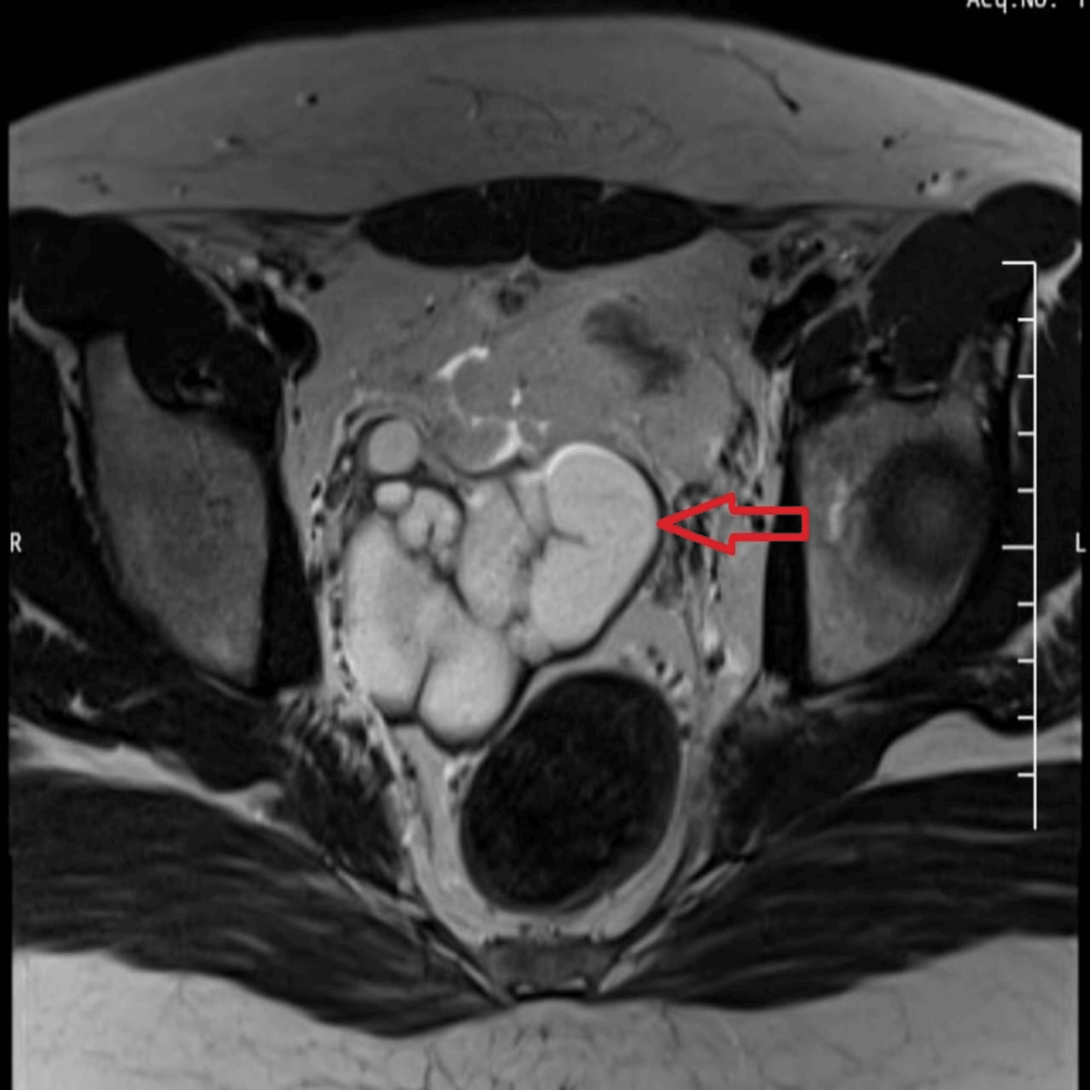
Axial T2-weighted MRI pelvis image reveals a hyperintense, tubular seminal vesicle cyst with incomplete internal septations adjacent to the prostate (arrow). MRI, Magnetic resonance imaging

**Figure 4:**
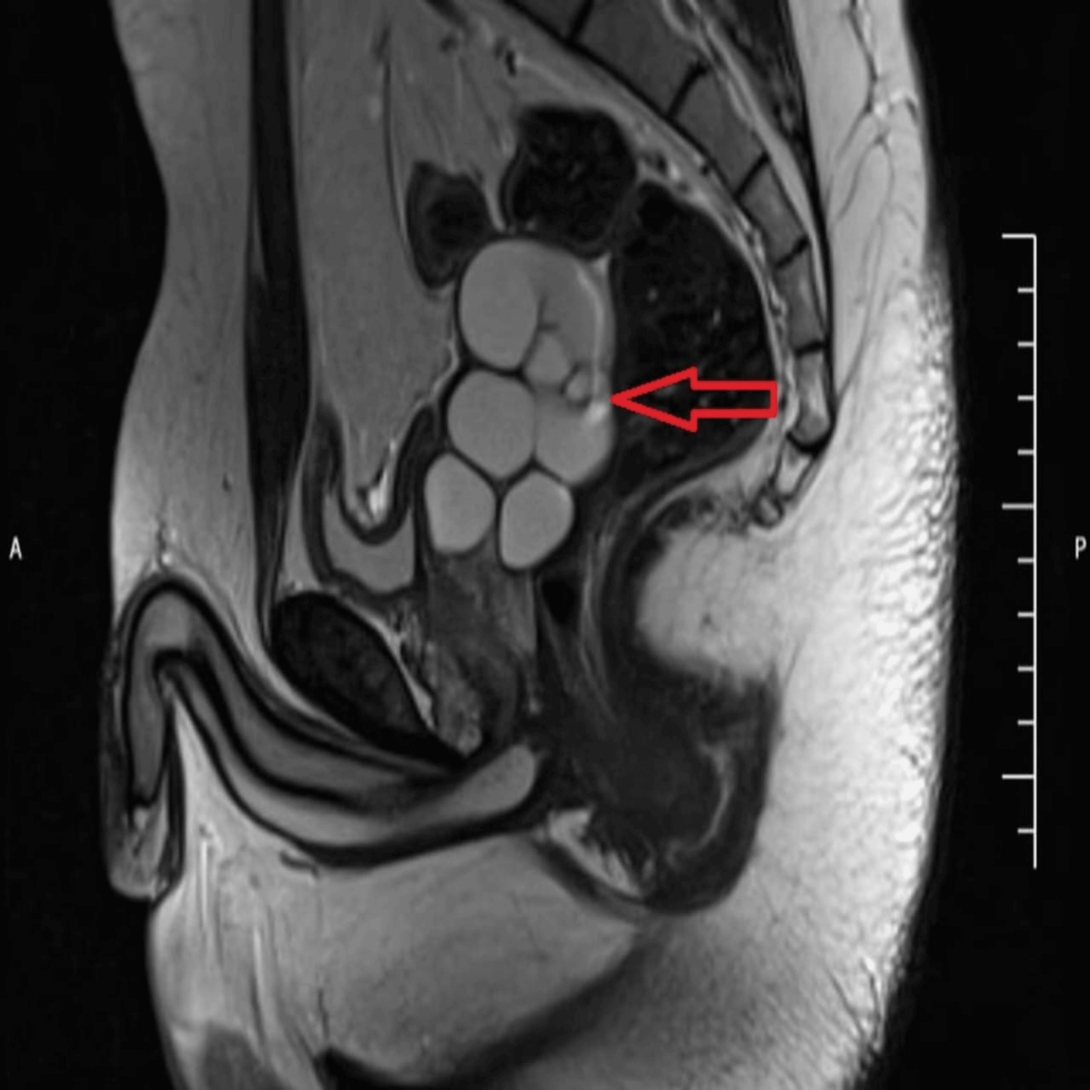
Sagittal T2-weighted MRI pelvis image reveals a hyperintense, tubular seminal vesicle cyst with incomplete septations adjacent to the prostate in the retrovesical space (arrow). MRI, Magnetic resonance imaging

**Figure 5:**
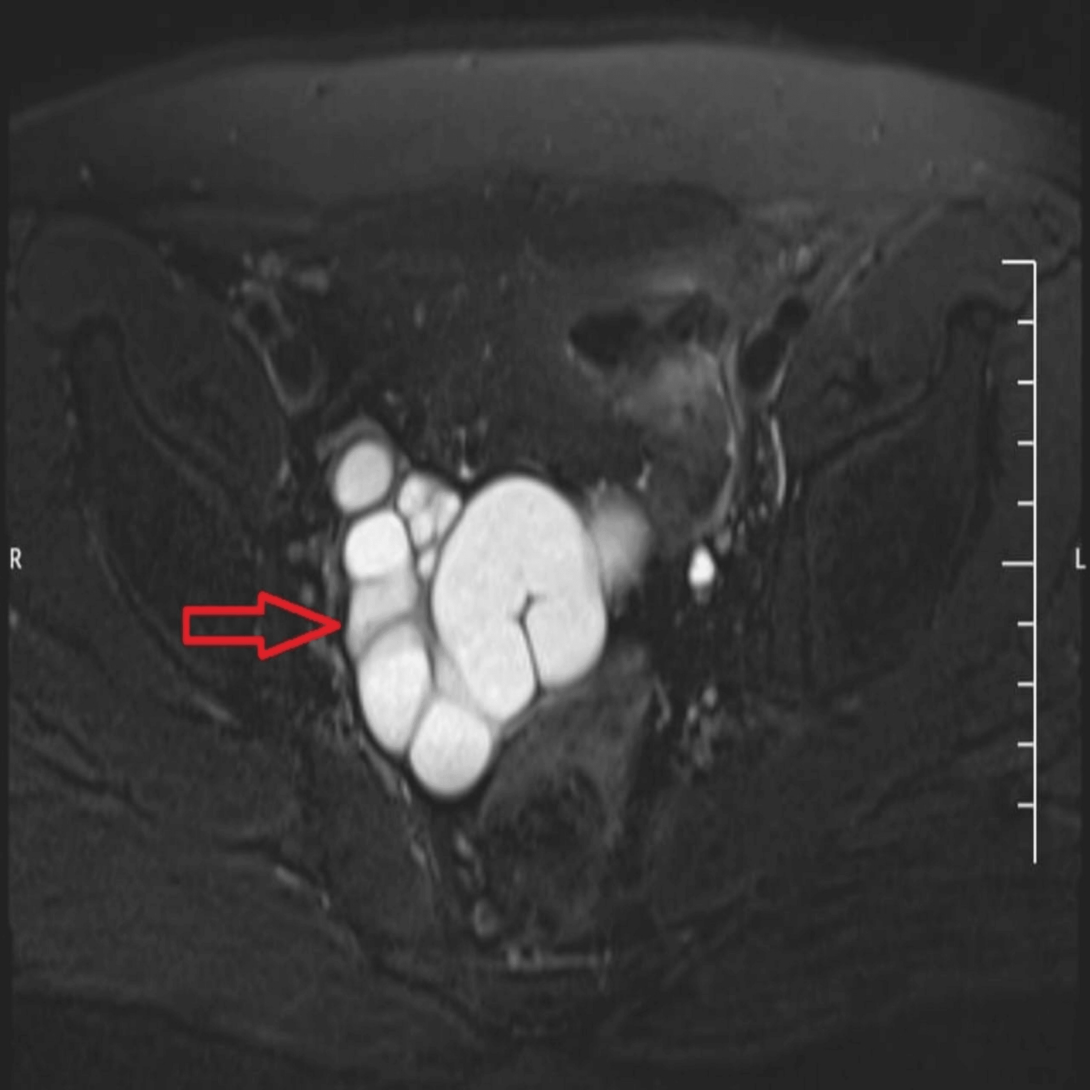
Axial STIR MRI pelvis image reveals a hyperintense, tubular cystic lesion with incomplete internal septations adjacent to the prostate, consistent with a seminal vesicle cyst (arrow). STIR, Short tau inversion recovery; MRI, Magnetic resonance imaging

Pelvic MRI confirmed a hyperintense tubular cystic structure on T2-weighted (Figures 3-4) and Short Tau Inversion Recovery (STIR) sequences (Figure 5), located adjacent to the prostate and seminal vesicle. The lesion exhibited simple fluid content, with no signs of hemorrhage or infection. The bladder, prostate, and contralateral seminal vesicle appeared normal. These imaging findings established the diagnosis of Zinner syndrome.

Our patient was offered minimally invasive surgical excision due to persistent symptoms and cyst size. He underwent successful laparoscopic seminal vesiculectomy, with uneventful recovery.

## Discussion

Zinner syndrome, first described by Zinner in 1914, is a rare congenital anomaly characterized by a classical triad: ipsilateral renal agenesis, seminal vesicle cyst, and ejaculatory duct obstruction [1,2]. This syndrome represents the male counterpart of Mayer-Rokitansky-Küster-Hauser syndrome in females. It arises due to maldevelopment of the mesonephric (Wolffian) duct during embryogenesis, which gives rise to the epididymis, vas deferens, seminal vesicles, and part of the ureteric bud. Disruption in this developmental cascade leads to the manifestations observed in Zinner syndrome [5]. During the 4th to 13th weeks of embryonic development, the mesonephric duct gives rise to the ureteric bud and the structures of the male reproductive tract. An insult or failure in the development of the ureteric bud leads to renal agenesis, while persistent distal mesonephric duct derivatives result in seminal vesicle cysts and ejaculatory duct obstruction. These anomalies typically remain asymptomatic until adolescence or adulthood, when increased seminal fluid production, hormonal changes, and a stenosed duct system prevent appropriate drainage, unmasking the symptoms [5-7]. Presentations include perineal discomfort, painful ejaculation, dysuria, epididymitis, prostatitis, or infertility [3-5].

Imaging plays an indispensable role in the diagnosis of Zinner syndrome. While transabdominal and transrectal ultrasonography can identify pelvic cysts, their limited resolution often necessitates cross-sectional imaging. CT urography is valuable for confirming renal agenesis and visualizing cystic lesions. MRI, with its superior soft tissue characterization and multiplanar capabilities, is the modality of choice for delineating seminal vesicle anatomy, assessing cyst contents, and ruling out differential diagnoses [6-8]. MRI can also detect associated anomalies, like ureteral ectopia, that are positioned more laterally [7,8].

Our case aligns with the literature in terms of delayed presentation, and the imaging findings were consistent with other reported cases of Zinner syndrome. Differential diagnoses include Müllerian duct cyst, ejaculatory duct cyst, prostatic utricle cyst, and ureterocele (Table 1). Müllerian duct cysts are midline structures that do not usually associate with renal anomalies. Ejaculatory duct cysts are located near the verumontanum and may present similarly. Prostatic utricle cysts are smaller and often midline, while ureteroceles are associated with duplicated systems [6-8].

**Table 1:** Differential diagnosis. MRI, Magnetic resonance imaging; CT, Computed tomography

Condition	Renal Agenesis	Seminal Vesicle Cyst	Preferred Imaging
Zinner syndrome	Yes	Yes	MRI, CT
Müllerian duct cyst	No	Yes	MRI
Prostatic utricle cyst	No	No	MRI
Ejaculatory duct cyst	No	Yes	MRI

Histopathologically, seminal vesicle cysts in Zinner syndrome are lined by cuboidal or columnar epithelium and may exhibit chronic inflammation. If secondarily infected, they may present with abscess formation. Cyst contents typically include seminal fluid, and chronic obstruction can result in backpressure changes and infertility. Zinner syndrome has important implications for reproductive health. Ejaculatory duct obstruction and altered seminal vesicle function may lead to oligospermia or azoospermia, causing infertility. Therefore, in adolescents and young adults, early diagnosis and timely surgical correction are essential to preserve fertility. 

Management of Zinner syndrome depends on the size of the cyst and symptomatology. Asymptomatic patients with small cysts may be managed conservatively, with periodic follow-up. In symptomatic cases, surgical intervention becomes necessary. Minimally invasive approaches, including laparoscopic or robot-assisted excision of the seminal vesicle cyst, have become the standard of care owing to reduced morbidity and excellent anatomical visualization. Other therapeutic options include transrectal or transperineal aspiration, cyst marsupialization, and transurethral unroofing, though these may carry a higher risk of recurrence [3,5,9]. Antibiotic prophylaxis is indicated in cases complicated by infection.

## Conclusions

Zinner syndrome, though an infrequent congenital anomaly, carries significant clinical implications when symptomatic. Accurate and early recognition - primarily through cross-sectional imaging such as MRI and CT urography - is crucial for timely intervention and preservation of reproductive function. Radiologists play a pivotal role in its identification, especially in young males with unexplained pelvic symptoms. Clinicians must remain vigilant to the subtle, often nonspecific presentation and should consider Zinner syndrome in the differential diagnosis. Early recognition can prevent complications related to cyst enlargement or infection. Advances in minimally invasive surgical techniques have further improved patient outcomes, offering definitive management with minimal morbidity. As our understanding of this condition evolves, a multidisciplinary approach involving urology, radiology, and reproductive medicine will remain central to optimizing care and ensuring a favorable long-term prognosis.
